# Language models can identify enzymatic binding sites in protein sequences

**DOI:** 10.1016/j.csbj.2024.04.012

**Published:** 2024-04-30

**Authors:** Yves Gaetan Nana Teukam, Loïc Kwate Dassi, Matteo Manica, Daniel Probst, Philippe Schwaller, Teodoro Laino

**Affiliations:** a IBM Research Europe, Saümerstrasse 4, 8803 Rüschlikon, Switzerland; b National Center for Competence in Research-Catalysis (NCCR-Catalysis), Switzerland

## Abstract

Recent advances in language modeling have had a tremendous impact on how we handle sequential data in science. Language architectures have emerged as a hotbed of innovation and creativity in natural language processing over the last decade, and have since gained prominence in modeling proteins and chemical processes, elucidating structural relationships from textual/sequential data. Surprisingly, some of these relationships refer to three-dimensional structural features, raising important questions on the dimensionality of the information encoded within sequential data. Here, we demonstrate that the unsupervised use of a language model architecture to a language representation of bio-catalyzed chemical reactions can capture the signal at the base of the substrate-binding site atomic interactions. This allows us to identify the three-dimensional binding site position in unknown protein sequences. The language representation comprises a reaction-simplified molecular-input line-entry system (SMILES) for substrate and products, and amino acid sequence information for the enzyme. This approach can recover, with no supervision, 52.13% of the binding site when considering co-crystallized substrate-enzyme structures as ground truth, vastly outperforming other attention-based models.

## Graphical abstract

**Figure:**
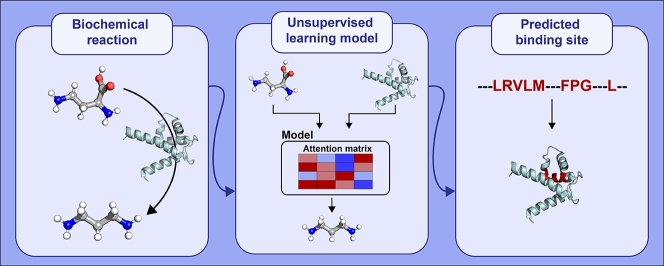

## 1 Introduction

Language Models (LMs) (e.g. BERT [1], GPT [2], and ELMo [3]) made the headlines being worldwide relevant for tasks such as information retrieval [4], text generation [5], [6], [7], and speech recognition [8]. The ability of LMs to learn a probability distribution over sequences of words in relation to a domain-specific language is the primary factor contributing to their success. In essence, these architectures encode distinct vector representations (embeddings) of a word based on its context, uncovering linguistic relationships between the different words of the domain-specific language.

Large Language Models (LLMs) [9], [10] trained on massive and diverse corpora are demonstrating critical new abilities, from writing innovative content [11] to resolving simple math problems [12]. These models achieved relatively high performance on new tasks for which they were not explicitly trained (also known as zero-shot learning tasks) [13], [14], most likely because the ability of language architecture to generalize on new tasks is the result of an unintentional multitask learning process. [14].

LMs, especially transformers and their derivatives (e.g. BERT [1], ALBERT [15], RoBERTa [16], etc.), have also had a relevant impact on chemistry and biology, reaching state-of-the-art performance when fine-tuned on specific tasks [17], [18], [19], [20], [21]. In chemistry, reagents, substrates, and products are usually depicted using a text representation such as SMILES (simplified molecular-input line-entry system) [22], [23]. Using this domain-specific representation, scientists have shown that LMs can learn to accurately map atoms between precursors and products with an unsupervised masked language modeling (MLM) [24], or predicting molecular properties using a BERT-like model trained in a semi-supervised way [25]. With the extension of string-based representations to proteins, LMs can be used for uncovering hidden relationships in biological tasks, such as predicting mutational effect, secondary structure [26], long-range contact prediction [27], binding site targeting [28], capturing important biophysical properties governing protein shape [28], [66], modeling bacterial activity [29], or predicting peptide binding [30].

The identification of binding and active site residues and the characterization of the corresponding protein function [31], [32], [33] pose a significant scientific challenge, particularly following groundbreaking research on protein structure prediction [20], [34]. The activity of a protein is directly correlates with the structure of its binding site [35], a spatial region hosting a contiguous or non-contiguous amino acid (AA) sequence. This region has evolved to facilitate selective interactions with specific molecules. The conservation of amino acids in the binding site over the entire sequence during evolution underscores their crucial role in providing unique structural features for enzyme function [36], [37]. Learning the signal describing the 3D interaction of amino acids with the target molecules in the binding site from the AA sequence and the molecular representation could enable the prediction of protein function derived from co-homology strategies [32], [38], [39], [40], [41], [42], [43], or protein-protein interaction networks [44]. Tools like Pfam [45] and PSI-BLAST [46] can help infer active site location based on sequence similarity or preserved domains information. Other approaches, such as those proposed by Zhang et al. [47] and Pande, Raheja, and Livesay [48], leverage machine learning methods like support vector machines (SVM) [49] and multilayer perceptron (MLPs) [50] for catalytic site prediction.

Building on Schwaller's work [24], we demonstrate that Language Models (LMs) can capture the signal characterizing AA binding sites using linguistic representations for proteins and their molecular substrates. We leverage a publicly available dataset of enzymatic reactions, where substrate molecules are represented with SMILES notation and proteins with their AA linear sequences. Unsupervised training can identify up to 52.13% of binding sites when considering co-crystallized substrate-enzyme structures as ground truth.

**Fig. 1:**
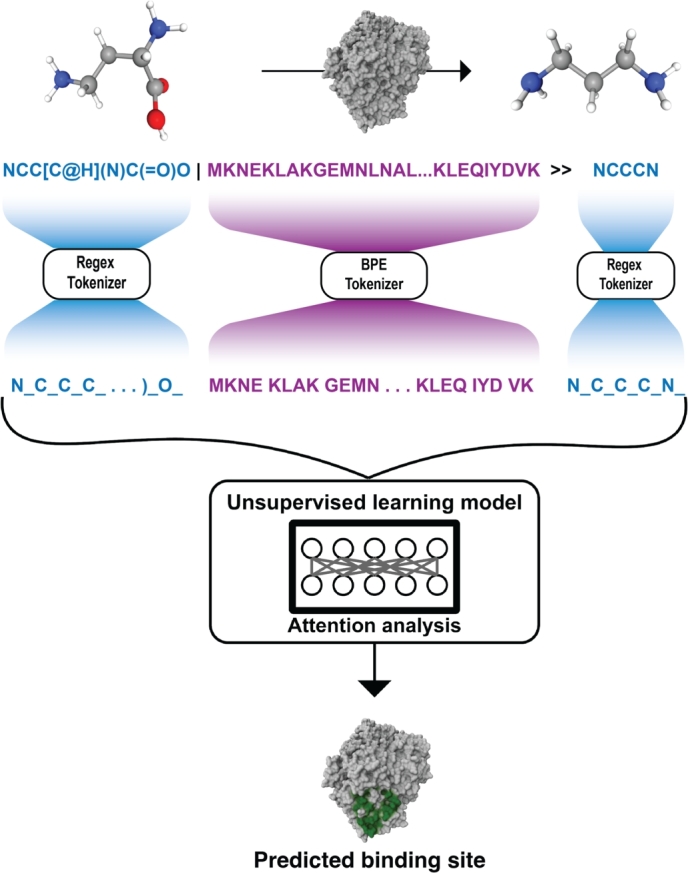
**RXNAAMAPPER pipeline**. A BERT model [1] is trained on a combination of organic and enzymatic reaction SMILES using MTL [52], leveraging atom-level tokenization and MLM [53] for the SMILES components, while Byte Pair Encoding (BPE) tokenization and n-gram MLM for the amino acid sequence part. The trained model is used in inference to define a score, based on the attention values computed on the reaction SMILES provided as input, which allows the prediction of the binding site of the enzyme bio-catalyzing the reaction with no supervision or structural information. The binding sites are represented in our plot as red regions in the molecule.

Inspired by the work of Schwaller et al. [24], here we show that LMs can capture the signal characterizing AA binding sites using a linguistic representation for proteins and their molecular substrates (see Fig. 1). We leverage a publicly available dataset of enzymatic reactions [51], where substrate molecules are represented with SMILES notation and proteins with their AA linear sequences. Unsupervised training can identify 52.13% of binding sites when considering co-crystallized substrate-enzyme structures as ground truth.

## 2 Methods

### 2.1 Dataset

We consider a dataset of 1 million organic reactions from USPTO [54], combined with a dataset of bio-catalyzed reactions called ECREACT [51]. The USPTO covers a wide range of chemical reactions, represented as reaction SMILES. This dataset is commonly employed in various applications within the field of chemistry, particularly in the development and training of machine learning models for reaction prediction and analysis due to its accessibility and extensive coverage of organic chemical reactions [24], [55], [56], [57]. On the other hand, ECREACT contains 62,222 reactions with a unique reaction-EC combination. The entries from this dataset can be classified based on their Enzyme Commission numbers (EC numbers) and grouped as shown in Table 1. ECREACT is the result of the combination of bio-catalyzed reactions from four databases: Brenda [58], Rhea [59], PathBank [60], and MethaNetX [61]. It predominantly features transferase-catalyzed reactions (EC 2.x.x.x), making up around 53.5% of entries due to the inclusion of non-primary lipid pathways from PathBank. Oxidoreductases and hydrolases account for 24.5% and 10.7% respectively, with lyases, isomerases, ligases, and translocases making up the rest. To align with the original ECREACT dataset, we maintained consistent data splits based on products to prevent bias towards specific reaction products during model training. By stratifying data using unique SMILES strings for products, we mitigate biases effectively. While removing duplicate reactions, we retained duplicated sequences or SMILES to expose the model to diverse variations and enhance learning robustness. Prior to data augmentation, our dataset contained 70.8% unique substrate SMILES and 57.9% unique product SMILES.

**Table 1:** ECREACT dataset division divided into groups based on the level of information.

Groups	N^o^ of reactions	Level of information
No EC number	55,115	No information about the enzyme
EC-level 1 (EC1)	55,707	Enzyme class
EC-level 1-2 (EC2)	56,222	EC1 + Subclass
EC-level 1-3 (EC3)	56,579	EC2 + sub-subclass
EC-level 1-4 (EC4)	62,222	EC3 + serial number in the subclass

### 2.2 Data processing

Using EC numbers as the single filter, we mapped the EC numbers to their corresponding AA sequences from Uniprot [62]. To enrich the training data with additional protein context, we constructed “augmented” reactions for each successful mapping. In these augmented reactions, we kept the original reactants and products from the ECREACT entry, but replaced the EC number with the corresponding retrieved protein sequence. Essentially, this transformed the data representation from a functional classification (EC number) to a more detailed protein sequence-based representation. A filtering step is performed to reduce the overrepresentation of certain EC numbers by limiting the maximum number of sequences per EC number to 10K. In cases where the number of sequences exceeded the limit for a particular EC number, we randomly selected 10K sequences from the set. While the data augmentation approach successfully enriched the training data with additional protein context, our analysis revealed that 96% of the protein sequences in the dataset are unique. Notably, the uniqueness of protein sequences stands out, influenced by the prevalence of certain reactions in datasets like USPTO, emphasizing popularity and organic chemistry biases.

### 2.3 Tokenization

Language models operate on numerical data, requiring text transformation into a numerical representation. An important step in the conversion is the tokenization step, which involves dividing a text into its constituent parts. Two different tokenization approaches are used to deal with the dual representation of the molecular entries in our dataset (SMILES and amino acids sequences). For SMILES, we use a Regex-based transformation [63] (character-level tokenization). The selection of the tokenizer for amino acids sequences is more complex. The complexity derives from: (a) the length of the sequences and (b) the limited number of input tokens supported by LMs. To overcome these limitations, we compressed our input sequences. We trained several tokenizers with various settings to maximize the sequence compression using a grid search over sequence length and vocabulary size.

All the tokenizers trained are Byte-Pair Encoding (BPE) tokenizers. To investigate the effect of the vocabulary size on the tokenizers' compression abilities, we set this variable to 10K, 20K, 30K, 50K, and 75K. We created subsets contained sequences falling within specific length intervals, including 250-900, 400-500, 600-750, and 900-1K amino acids. Each dataset consists of 400K sequences. By adopting this approach, we aimed to maintain the diversity of our search space while accommodating the unique characteristics of sequences across various length ranges. Additionally, to ensure comprehensive coverage and representation within our training data, we ensured to sample sequences from all EC classes present in the dataset.

### 2.4 Models and training procedure

Herein, we consider different transformer architectures, i.e., BERT [1] and Albert [15], exploring various approaches: training from scratch, fine-tuning, and pre-trained models.

In training, to better control the token masking and handle the different lengths of the enzymatic reaction components, the model variants have been jointly optimized with Masked Language Modeling (MLM) and an n-gram Masked Language Modeling [53] (n-gram MLM, by randomly masking out 15% of the input tokens). MLM and n-gram MLM have been applied to substrates/products and enzymes respectively. As the models are trained on different datasets, i.e. ECREACT [51] and USPTO [54], Multi-task Transfer Learning (MTL) [52] has been adopted on a combination of reaction SMILES representing organic reactions (weight assigned 0.1) and bio-catalyzed reactions (weight assigned 0.9) to create a task-specific language model able to understand bio-catalyzed reactions.

The use of USPTO in a transfer learning process is to ease the understanding of generic chemistry and SMILES syntax. For enzymatic reactions, each example consists of a reaction SMILES complemented with the AA sequence representation of the enzyme of interest (see Fig. 1 for a depiction). As we train the model via MLM and n-gram MLM, we sparsely mask the reactants and the products and densely mask the enzyme sequence.

Six million (6M) reactions subset of the preprocessed ECRACT was chosen at random and used as the training set for all language models. Another 2.5M ECREACT subset was chosen as the validation set to compute the validation loss. For all the language models we used default hyper-parameters from the HuggingFace implementation. As an optimizer we adopted ADAM [64] for 50,000 training steps.

It has been recently shown that large pre-trained models on natural language can be fine-tuned on different data modalities to attain comparative performance with respect to models trained on downstream tasks [65]. Inspired by this seminal work, here we also decided to include in our study the following models: ProtAlbert [66] and ProtBert [66] (pre-trained on protein data and fine-tune on biocatalyzed data), BERT-base (pre-trained on various data modalities), BERT-base [1] and BERT-Large [1] (trained from scratch on biocatalyzed data).

### 2.5 Binding site prediction

The prediction of the binding regions of proteins is unsupervised and entirely based on the analysis of the attention values computed by the pre-trained language model after encoding a reaction. If we label $S\in R^{l\times d}$ (l=r+m+p) as the embedding of a given reaction and *r*, *m*, and *p* refer to the length of the reactants, the enzyme, and the products, respectively, a forward pass of *S* through the model yields a sequence $S^{\prime }$ with the same dimension as *S*. Each encoder block computes the attention matrix $A\in R^{l\times l}$ of the sequence *S* provided as input [67]. We construct a matrix $P\in R^{r\times m}$ by summing two sub-matrices of *A*, describing the link between reactants and enzymes: $P=A[1:r,1:m]+A{\left[r+1:r+1+m,1:r\right]}^{T}$. We use the matrix *P* as shown in the Algorithm 1 to predict the binding regions via a consensus scheme where each reactant's atom has *k* votes to choose its best-bound enzyme's token. The selected enzyme's tokens are uniquely gathered in a set and are considered the protein's binding region. Hereinafter, the method combined with BERT-base and the BPE will be referred to as RXNAAMapper.

**Algorithm 1:**
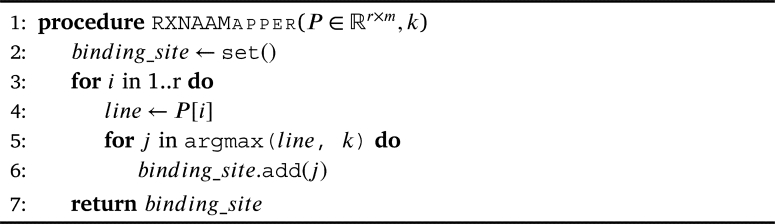
Binding site prediction.

### 2.6 Evaluation

We use a set of 777 co-crystallized ligand-protein pairs from the Protein-Ligand Interaction Profiler (PLIP) [68] as ground-truth, to perform a two-fold evaluation: (1) a sequence-based assessment benchmarking RXNAAMapper against two fine-tuned protein language models (ProtAlbert and ProtBert), a statistical baseline (Random Model), two pre-trained BERT models on natural language (BERT-base and BERT-Large) and the alignments retrieved from Pfam [45]; (2) a structural validation with protein-ligand binding energies computed with docking. We used Pfam annotations for a fair assessment with existing methods using sequence information only. For the sequence-based evaluation, we use an overlap score between the prediction and the ground truth, as well as the false positive rate. The overlap score is defined considering the binding site as a set of non-overlapping segments in a sequence. If *S* with |S|=n is a sequence of amino acid residues, the binding region $B_{s}$ of *S* is defined as $B_{s}={\left\{(a_{i},b_{i})\right\}}_{i}^{m}$, where $a_{i}$ and $b_{i}$ are the index boundaries of the segment *i*. The overlap score ($OS(B,B_{s})$) between the predicted binding region $B={\left\{(a_{pi},b_{pi})\right\}}_{i}^{n}$ and the ground-truth $B_{s}={\left\{(a_{si},b_{si})\right\}}_{i}^{m}$ is defined as:

$$OS(B,B_{s})=\frac{\sum_{i}^{n}\sum_{j}^{m}\max(0,\min(b_{pi},b_{sj})-\max(a_{pi},a_{sj}))}{\sum_{i}^{m}(b_{si}-a_{si})}$$

Besides the overlap score, the false positive rate (*FPR*) of the predictions is defined as:

$$FPR=\frac{\sum_{i}^{n}(b_{pi}-a_{pi})1_{\overset{m}{\underset{j=1}{⋀}}[a_{pi},b_{pi}]\cap [a_{sj},b_{sj}]=\emptyset }}{\sum_{i}^{n}(b_{pi}-a_{pi})}$$

For the structural assessment, on a set of 2213 protein-ligand binding site predictions, we evaluated the binding energy computed with Autodock Vina [69], [70] considering predicted binding sites and the ground truth from PLIP. We chose these enzyme-ligand pairs by first matching PDBs and amino acid sequences with annotated binding sites from PLIP. Then filtering reactions catalyzed by enzymes not present in our training set. We then selected the reactions having unique combinations of PDB, EC number, ligands, and predicted binding sites. We computed the Cartesian coordinates of the ligand and receptor molecules, which are generally retrieved from the PDB [71] or PDBQT [72] for the protein, and PDB, PDBQT, or Mol2 for the ligand. To calculate the binding free energy of a ligand to an enzyme, we first computed a grid box centered on the binding site where the ligand is to be docked. The box has been found by averaging the 3D coordinates of the atoms of the binding site and setting the box side length to 50 Å.

## 3 Results

### 3.1 Sequence compression and representation

Before language modeling, amino acid sequences must be numerically encoded using an encoding scheme that gives each amino acid sequence a unique vector representation. This encoding method acts as a map from the input amino acids to a point in the protein representation space. The embedding should be able to capture key features of each element (also called a token) that is encoded and should be able to preserve the relationship between the encoded elements (typically expressed geometrically through a vectorial representation of the encoded tokens).

Here we consider a Transformer model based on BERT that in a standard setup handles a maximum of 512 input tokens to generate the encoding vector. For architectures like ours, 512 input tokens size is a pretty strict limitation due to the large memory footprint coming from self-attention layers, whose complexity scales quadratically with the input sequence length [73]. This quadratical growth in complexity translates to a dramatic increase in processing time, contrasting the model's ability to analyze the input text in a timely manner. In protein modeling, the model must see the entire amino acid sequence in order to learn crucial structural information. Given that amino acid sequences can be prohibitively long (see Figure S2, finding a good compression and representation scheme becomes a fundamental task before the model training.

**Fig. 2:**
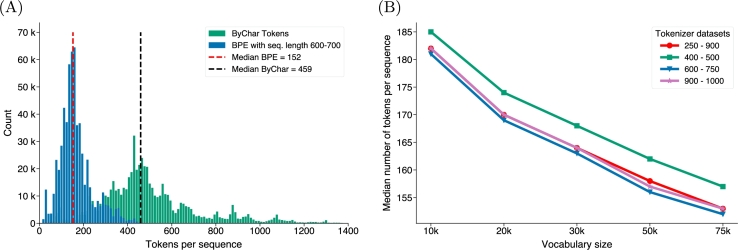
**BPE analysis**. The left plot displays the density distribution of token counts obtained using the BPE tokenizer, contrasting it with the ByChar tokenizer used in our model training. Unlike ByChar, BPE splits infrequent fragments into two or more tokens while merging the most frequent ones into longer tokens. This application leads to a sequence shortening effect compared to ByChar tokenization. On the right, the median of BPE token counts is shown for each configuration in our grid search. The results indicate that larger vocabularies lead to more significant data compression.

We trained different Byte-Pair Encoding (BPE) tokenizers with various settings (as described in the method section) to find the set of parameters that maximizes the compression of the amino acid sequences in terms of vocabulary size and sequence length. The compression power of the tokenizers trained has been tested on a dataset of random sequences from Uniprot (n=600K). Fig. 2B shows a negative correlation between the vocabulary size and the median number of tokens for the same dataset. This result confirms that by increasing the vocabulary size we are implicitly increasing the length of BPE tokens in our vocabulary, as we merge the most frequently occurring fragment of sequences into single subwords or fragments. When analyzing the impact of vocabulary size and sequence length, we observed that sequences in the training set ranging from 600 to 700 amino acids, paired with a vocabulary size of 75K, achieved optimal compression with a median token count of 152 (see Fig. 2A and Table S1). This represents a compression rate of 66.8% compared to the baseline character-level tokenizer (ByChar) which tokenizes sequences into individual amino acids and had a median token count of 459. Sequences within the 600-750 amino acid range strike a balance between capturing sequence information effectively and keeping input lengths manageable for language model processing. This range enables efficient compression by the tokenizer while retaining essential details. The use of a 75k vocabulary size allows the tokenizer to cover a wide range of amino acid combinations and rare subword structures, enhancing its ability to represent diverse sequences accurately and contribute to effective compression. Therefore by using this tokenization scheme, we overcome the architectural limitations and train our model on broader corpora.

We rely on two distinct tokenization methods for the AA sequences and the molecular substrates (SMILES). We applied BPE only to AA sequences to compress the resulting token sequence length. On the other hand, the SMILES strings representing substrates naturally allow for tokenization at atoms and bonds level resulting in relatively short token sequences, making extensive compression unnecessary. Therefore we opted for a regular expression-based tokenization approach [55], [63].

### 3.2 Binding site prediction

Enzyme binding sites are areas on an enzyme's surface specifically intended to interact with other molecules. Enzymes can have many types of binding sites that perform distinct tasks and engage different molecules. The most significant is the active site, which includes catalytic residues to carry out the enzymatic reaction on a substrate. We trained the RXNAAMapper (a BERT-base model combined with BPE tokenization on the amino acid sequences developed in this work), BERT-base, BERT-Large, ProtAlbert, and ProtBert (details in methods) and compared them on the task of binding site predictions using 777 amino acid sequences from PLIP (with related binding sites) as ground truth. The predictions are based on the self-attention analysis of the models. Self-attention modules, a key component in Transformers-based models, is an attention mechanism that connects distinct points in a single sequence to calculate a representation of the same sequence. To match the required output dimension, the separate attention ‘heads’ are commonly concatenated and multiplied by a linear layer [67] forming a multi-head attention system. During our analysis we explored how three key factors influenced our model's performance in predicting binding sites. First, different attention heads prioritize different information within the sequence. We found that head 10 achieved the best results, suggesting that its focus on specific relationships was particularly relevant for this task Figure S5. Second, within this model paradigm, information flows through the layers sequentially, refining the model's abstraction. We observed that layer 5 provided the optimal level of context for binding site prediction. Finally, the model computed interactions between individual amino acids and reactant atoms. By focusing on the top 6 atoms, we achieved the most accurate predictions, indicating that capturing local interactions was key. The set of parameters giving the highest overlap between our predictions and ground truth is head=10, layer=5, and $top_{k}=5$.

For each trained model, we determined their performance by selecting the combination giving the highest overlap between the predictions and ground truth from PLIP (details in the methods section). We find that RXNAAMapper performs consistently better than the other unsupervised sequence-based methods. Among the binding sites predicted by RXNAAMapper, up to 52.13% overlap with the ground truth whereas ProtAlbert, ProtBert, BERT-base, BERT-Large, and the random model, reached 18.27%, 20.03%, 15.27%, 45.91%, and 14.07%, respectively predictions (Table 2). As a reference, we report the overlap score obtained by homology-based on Pfam annotations (67.37%).

**Table 2:** **Performance on sequence-based binding site prediction**. Reported in the table are the overlap score and the false positive rates for the binding site prediction using PLIP as ground truth for the seven methods considered: a random model, Pfam alignment-based model, a pre-trained BERT-base model, a pre-trained BERT-Large model coupled with a BPE tokenizer, ProtAlbert, ProtBert, and RXNAAMapper. Among these models, Pfam-based predictions are based on homology present within Pfam families. The others are attention based models extracted from unsupervised language models.

	Overlap Score	False Positive Rate
Random Model	14.07%	13.80%
BERT-base	15.27%	14.92%
BERT-Large + BPE	45.91%	42.71%
ProtAlbert	18.27%	16.23%
ProtBert	20.03%	16.42%
RXNAAMapper (ours)	52.13%	47.89%

Pfam-based	67.37%	61.68%

When comparing the performance of the sequence-based models, RXNAAMapper and BERT-Large + BPE, the Wilcoxon-rank test [74] revealed a statistically significant difference in the “Overlap Score” metric (p<0.001), where RXNAAMapper showed a higher overlap compared to BERT-Large + BPE.

Predictions based on homology models, like the one obtained using Pfam annotations, can help recover binding sites. However, these approaches use heuristics-based methods, giving rise to high frequencies of false positive rates (Pfam-based = 61.68%). The high false positive rate suggests that the area predicted as binding sites are too large. This might be attributed to the inclusion in the preserved domains of regions containing the active site, housing both the binding and the catalytic sites. However, active sites usually account for just 10-20% of the volume of an enzyme [75], while Pfam's-based approach predicted [45] on average 61.99% of the sequence as sites of interest. Our model predicted shorter stretches of the input sequences as binding sites (on average 48.06%) while maintaining a lower false positive rate (47.89%) compared to the homology-based.

We inspect the performance of our model and the Pfam-based across different enzyme classes (see Fig. 4A) and reaction classes (see Fig. 4B). Certain types of enzymes (e.g. Transferases) and reaction classes (e.g. functional group interconversion (FGI)) have a better compromise of overlap score and false positive rate with respect to other classes. Our model predictions differ from the homology-based ones, particularly in the case of Lysases. It demonstrates a more conservative approach by generating shorter predictions (on average 44.5 amino acids), leading to a lower false positive rate and overlap score. In contrast, Pfam-based predictions tend to be longer (on average 254 amino acids), contributing to a higher false positive rate. This discrepancy is linked to the limitations of sequence alignments in accurately predicting results when sequence identity falls below a specific threshold [76]. Conversely to alignment-based methods, our methodology (an alignment-free approach) captures evolutionary events without the assumption that homologous sequences are the consequence of a succession of linearly organized and more or less conserved sequence regions.

**Fig. 3:**
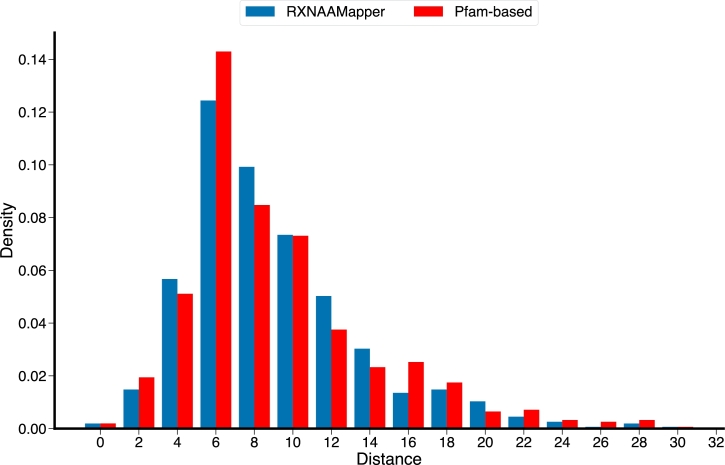
**Binding sites distance from ground truth**. Distribution plot depicting the distance of the predicted binding sites from the PLIP annotations. For both predictions, the distribution is right skewed reflecting the correctness of the predictions. RXNAAMapper exhibits a distribution peak at lower values, confirming the superior accuracy of its predictions in comparison to Pfam annotations.

We further compared our prediction and those from homology-based by looking at the distance between the barycenter of the grid boxes centered on the predicted binding sites and the ground truths. Although our model has lower overlap scores compared to the Pfam-based, our ability to control the false positive rate is reflected on the barycenter of our predictions to be spatially closer to the ground truth (see Fig. 3 and Figure S1).

**Fig. 4:**
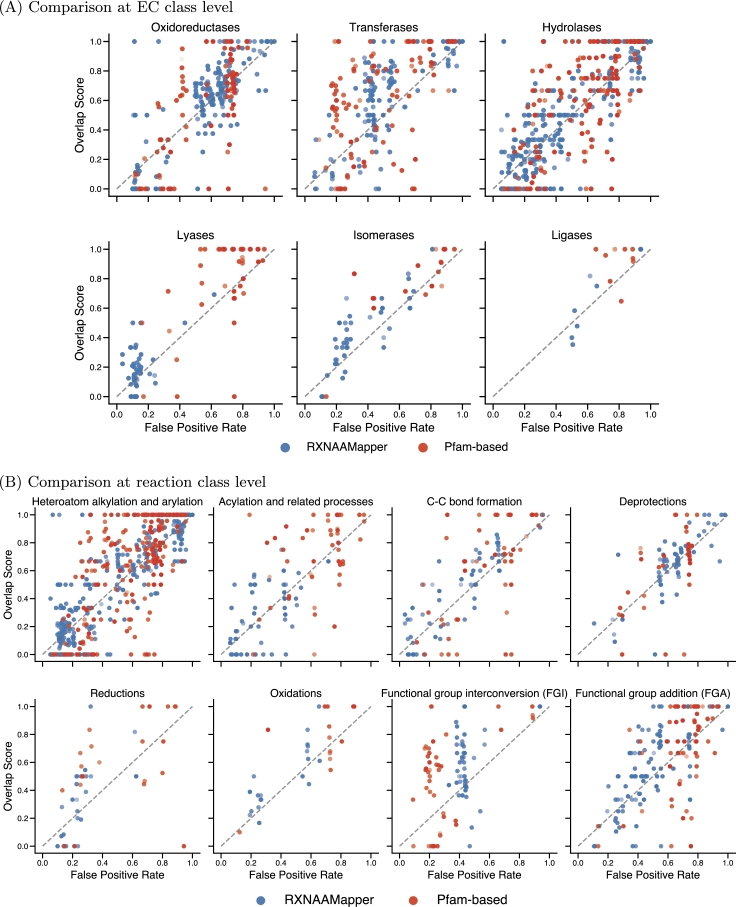
**Pfam-based and RXNAAMapper performances with respect to the EC classes and reaction classes**. The models exhibit different performances for different types of reactions and enzymes, underlying the modeling complexity of certain types of reactions and enzymes. For almost every enzyme class and reaction class, our model's predictions are on the bottom left of the figures (lower overlap score and false positive rate), while Pfam-based predictions are on the symmetrically opposite side of the figure (higher overlap and false positive rate). This highlights the ability of our model to predict binding sites while keeping the false positive rate within descent frequencies. The transparency in the figures correlates with the distance between the barycenter of the grid boxes centered on the predicted binding sites and the one centered on the ground truth. The closer the prediction is to the ground truth, the more opaque the point.

**Fig. 5:**
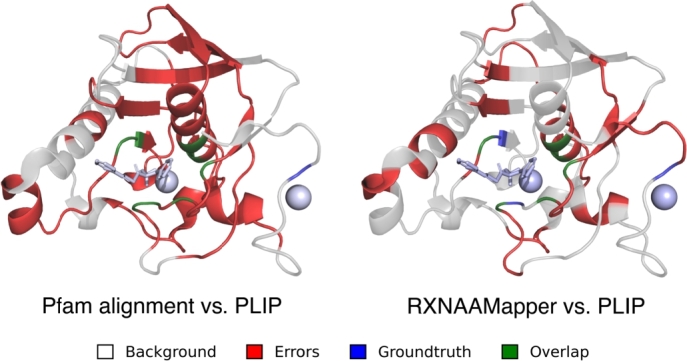
**Experiment results**. Comparison of the prediction from Pfam alignments (left) and RXNAAMapper (right) using PLIP as a ground-truth (PDB id: 6JFR) interacting with S-(2-oxo-2-phenylethyl) (2R)-2-benzyl-4,4,4-trifluorobutanethioate (K3U) and Nickel (II) ion). The area in red represents the predicted binding site region, while the blue area represents the ground truth of bindingsites. The white area depicts the backbone of the protein.

Fig. 5 shows an example of Pfam-based and RXNAAMapper predictions overlapped with the PLIP ground truth. Despite RXNAAMapper is covering fewer amino acids (35.9%) than the Pfam-based approach (66.3%), it demonstrated a recall rate comparable to Pfam-based predictions. Furthermore, the lower false positive rate observed in RXNAAMapper predictions (33.5% compared to 65.5% in Pfam-based predictions) suggests a more focused prediction of relevant amino acids, minimizing the inclusion of irrelevant ones. To complement our analysis, in the supplementary section we report a comparison of RXNAAMapper with a supervised approach showing competitive results (see Figure S3).

While RXNAAMapper did not surpass Pfam in terms of ground truth overlap, it offers distinct advantages in specific contexts, such as for Transferases and Lyases. Unlike homology-based methods, RXNAAMapper performs better even in scenarios where homologous sequences or functional annotations are scarce or absent, as observed for Lyase enzymes. This capability to generalize across different enzyme classes enables RXNAAMapper to navigate diverse biological contexts, contributing to its versatility and applicability beyond the limitations of homology-based approaches.

### 3.3 Structural validation

A successful chemical reaction hinges on the precise interaction between substrate and enzyme. The active site, delineated as a specific region on the enzyme comprising two crucial subdomains: the substrate-binding site and the catalytic site, fulfills distinct functional roles within the enzymatic mechanism. The binding site secures the substrate in position, while the catalytic site orchestrates the actual chemical transformation. This cooperation not only ensures specificity, but also drives the process by supplying energy to sustain substrate engagement.

The relation between enzyme and substrate within the active site relies heavily on the binding site's ability to hold the substrate in place and provide stabilizing energy. This specificity and stability are crucial for efficient chemical reactions. Therefore, accurately predicting binding sites becomes a key step in understanding and optimizing these interactions.

**Fig. 6:**
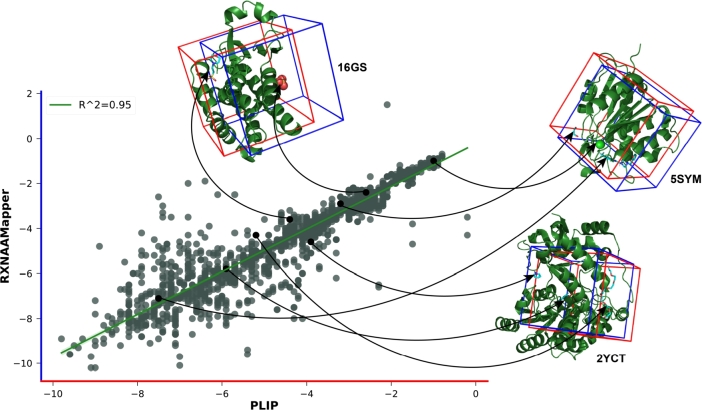
**Negative binding energy of 2213 enzyme-ligand pairs**. The figure shows how the energy scores deriving from the RXNAAMapper binding site predictions are in the same range with respect to those predicted from PLIP (R^2^=0.95).

To evaluate RXNAAMapper's ability to predict effective binding sites, we employed a docking approach. We tested our model on 2213 enzyme-ligand pairs (extracted from our dataset and the PLIP database - see Methods section) and compared the predicted binding energies (using Autodock Vina with RXNAAMapper's sites) with experimental data from PLIP. The results, with an average difference of only 0.37 kcal/mol, demonstrate RXNAAMapper's potential for accurate binding site prediction and its valuable contribution to understanding enzyme-ligand interactions (Fig. 6).

## 4 Discussion

The prediction of protein binding sites, which are critical and conserved functional areas of proteins, is crucial to improving our understanding of protein function. Moreover, the ability to detect these regions in an unsupervised fashion, solely relying on AA sequence information, allows an initial characterization of novel proteins.

Herein, we tackled the problem by introducing RXNAAMapper, a technology that uses pre-trained language models based on textual representations of biochemical reactions to identify binding sites in amino acid sequences. When tested on PLIP protein-ligand interactions, our approach outperforms other sequence-based methods by identifying more than 52% of proteins' binding regions with a lower false-positive rate. The combination of a model like a BERT-base and a BPE tokenization system leverage an as-of-now unexplored potential.

One of the main limitations in applying language models to enzymatic reactions is the computational burden introduced by handling long sequences [73], [77], [78]. Compressing the representations using efficient tokenization strategies mitigates the problem, but it has also the detrimental effect of discarding data points that may contain useful information. The use of a BPE tokenizer allowed us to train our model on entire amino acid sequences by compressing sequences in a lossless fashion. Leveraging the full sequence is a key component of our model as amino acids that in a protein sequence are far away may come together in the 3D representation. Unlike other models evaluated in this paper, RXNAAMapper demonstrated the ability to capture the syntax of bio-catalyzed reactions and grasp relevant features of the AA sequences by detecting regions of importance via reaction language modeling.

To further validate the predictions of RXNAAMapper, we evaluated the inferred binding sites of several enzyme-ligand pairs, achieving close agreement with experimentally determined sites (from the PLIP database). This ability to accurately identify binding sites is crucial, as it allows for modeling their interactions and predicting binding energies. Crucially, this analysis paves the way for predicting entire active sites. However, to fully evaluate the reconstruction of the entire active site, necessitates accurate annotations of catalytic sites due to their critical role in enzyme function.

Our method demonstrates superior control over false positives and is reflected on the barycenter of our predictions to be spatially closer to the ground truth. This represents a significant advancement as RXNAAMapper, unlike homology methods relying on evolutionary relationships, can predict binding sites for proteins with limited or no annotations. For future advancements in binding site prediction, exploring multi-modal deep learning model integration such as Mixtral 8x7B [79] and implementing fine-tuning strategies customized for biological sequences and interactions [80] are crucial steps to enhance predictive capabilities.

This work set a stepping stone towards novel in-silico approaches for protein function identification, and it is further evidence of the amazing ability of language models to retrieve 3D structure information from 1D sequential representation. We are confident that future language models with yet-to-be-unveiled capabilities will continue to offer innovative solutions to complex tasks using domain-specific languages without supervision.

## CRediT authorship contribution statement

**Yves Gaetan Nana Teukam:** Data curation, Validation, Visualization, Writing – original draft, Writing – review & editing. **Loïc Kwate Dassi:** Data curation. **Matteo Manica:** Conceptualization, Supervision. **Daniel Probst:** Data curation. **Philippe Schwaller:** Data curation. **Teodoro Laino:** Conceptualization, Supervision.

## Declaration of Competing Interest

The authors declare that there are no conflict of interest.

## Data Availability

The ECREACT data set is publicly available at the URL https://github.com/rxn4chemistry/biocatalysis-model. The code is available at the URL https://github.com/rxn4chemistry/rxnaamapper. Structures of docked proteins and results are available at the URL https://doi.org/10.5281/zenodo.7530180.

## Appendix A Supplementary material

MMC Additional table and figure on LM for binding sites prediction. MMC
