# Supplementary material for: Language models can identify enzymatic binding sites in protein sequences
Source: Comput Struct Biotechnol J. 2024 Apr 30;23:1929–37. doi: 10.1016/j.csbj.2024.04.012 (PMC11087710; doi:10.1016/j.csbj.2024.04.012)
Supplement: MMC — Additional table and figure on LM for binding sites prediction. [file mmc1.pdf]

Supporting Information

Language models can identify enzymatic active sites in protein sequences

Yves Gaetan Nana Teukam[1,*], Loïc Kwate Dassi[1], Matteo Manica[1], Daniel Probst[1,2], Philippe Schwaller[1,2], and Teodoro Laino[1,2]

[1]IBM Research Europe, Saümerstrasse 4, 8803 Rüschlikon, Switzerland

[2]National Center for Competence in Research-Catalysis (NCCR-Catalysis), Switzerland

[*]yna@zurich.ibm.com

April 25, 2024

List of Tables

1. Tokenizers training statistics 2
2. F1 score and Balanced Accuracy (BACC) on binding site prediction 2

List of Figures

1. Distance between the predicted active sites and ground truth 3
2. Frequency distribution of sequence length in training and test sets 4
3. Binding sites distance from ground truth 5
4. Performance of the model evaluated by Recall 6
5. Computational efficiency of RXNAAMapper compared to other language models 8

Vocabulary size	BPE datasets			
	**200-900**	**400-500**	**600-750**	**900-1K**
10K	182	185	181	182
20K	170	174	169	170
30K	164	168	163	164
50K	158	162	156	157
75K	153	157	**152**	153

Table 1: **Tokenizers training statistics**: Median number of tokens per sequence

	F1 score	BACC
Random Model	6.06%	50.13%
BERT-base	5.70%	50.18%
BERT-Large + BPE	7.70%	51.60%
ProtAlbert	6.56%	51.02%
ProtBert	7.21%	51.81%
RXNAAMapper (ours)	8.10%	52.12%
Pfam-based	8.73%	52.83%

Table 2: **F1 score and Balanced Accuracy (BACC) on binding site prediction**. Reported in the table are the F1 and BACC for the binding site prediction using PLIP as ground truth. All the models present a very low F1 score indicating the strugle of the models to balance precision and recall. This happens because of the high false positives. This high false positive come from the inherit imbalance characteristic of binding sites, as they represented a very small portion of the total sequence.

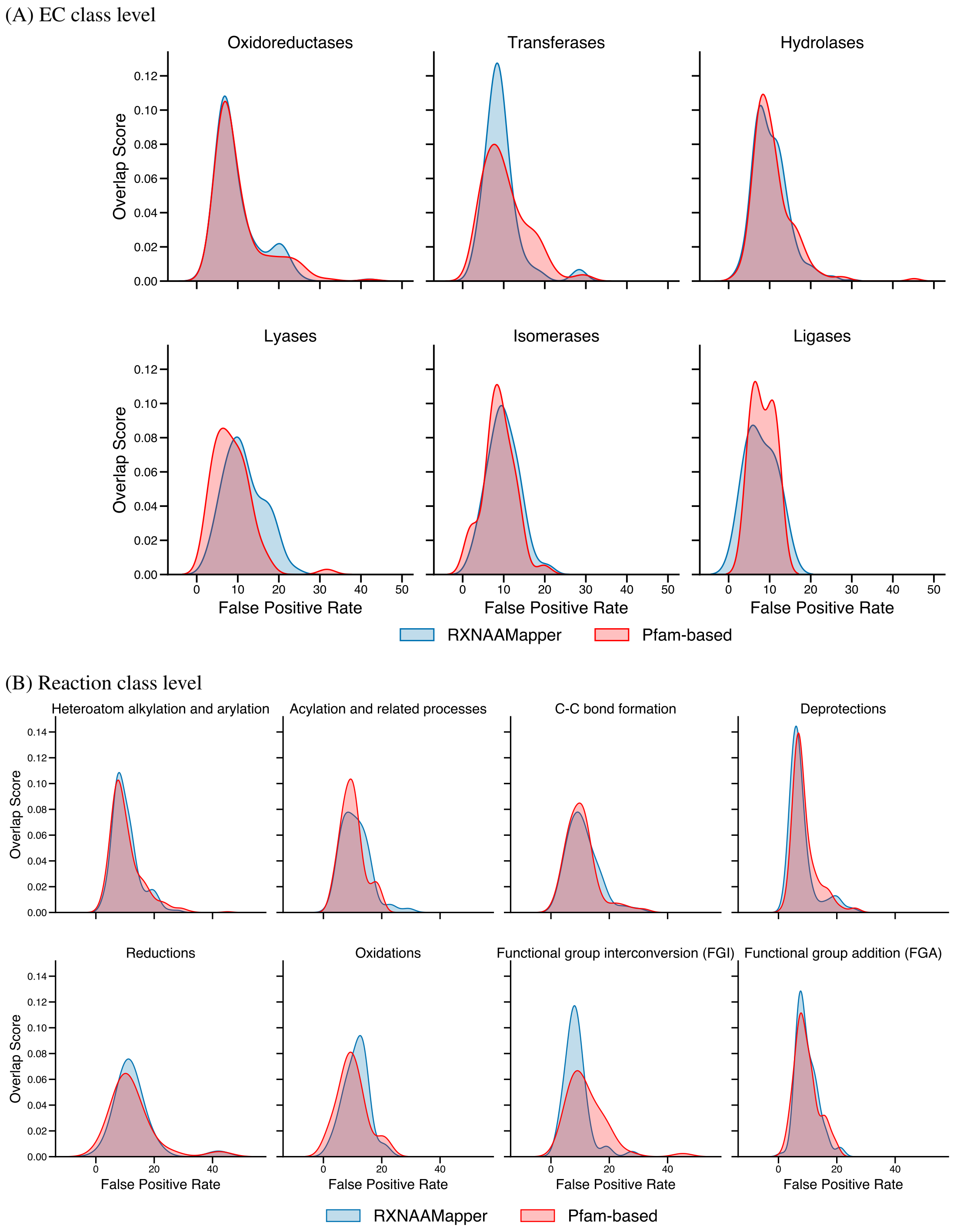

Figure 1: **Distance between the predicted binding sites and ground truth**. The distance between the barycenter of the grid boxes centred on the predicted binding sites and the ground facts was used to compare our prediction to those from the homology-based. (A) has been computed by grouping the points in out set by EC classes, while (B) on reaction classes.

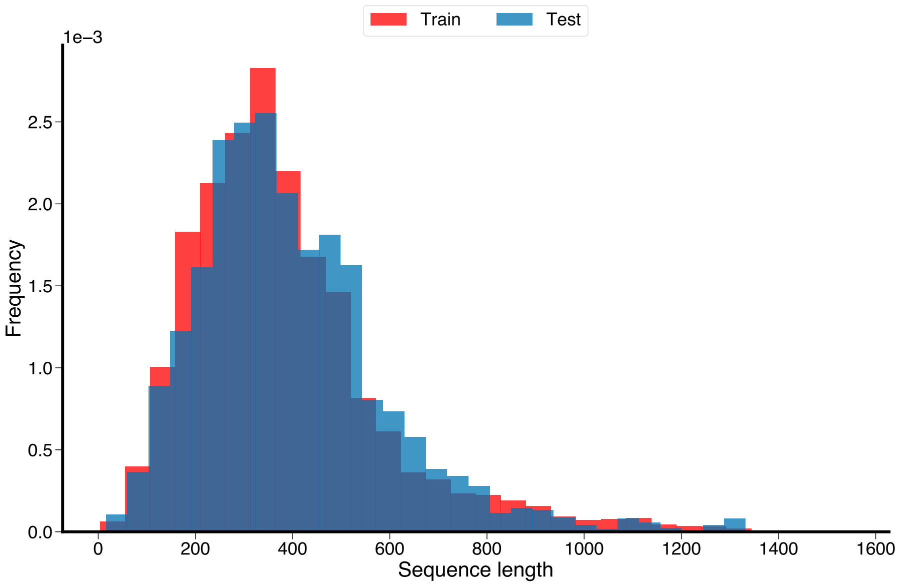

Figure 2: **Frequency distribution of sequence length in training and test sets**. This figure illustrates the frequency distribution of protein sequence lengths in the training and test sets. The distribution of sequence lengths in the test set closely matches the distribution in the training set, indicating that the test set is representative of the training data in terms of sequence length characteristics. During the training process, our model automatically filters out reactions that, when tokenized, have more than 512 tokens. From the remaining reactions, we extracted the sequences and noticed that we have many sequences with more than 512 amino acids. Without compressing these longer sequences using a Byte Pair Encoding (BPE) approach, we would have been left with only around 50% of the reactions for model training and evaluation. The use of BPE allowed us to effectively represent these longer sequences, enabling the model to learn from a more comprehensive dataset and improving its ability to generalize to unseen data. This frequency plot highlights the alignment between the training and test set distributions, demonstrating the successful incorporation of longer sequences (exceeding 1000 amino acids) in our datasets through the utilization of BPE.

Supervised approach vs. RXNAAMapper: We conducted a comparative study between a supervised methodology and RXNAAMapper. The supervised method leveraged token embeddings extracted from a ProtBert model and adopted the XGBoost algorithm as a predictive model to predict if a token is part of the binding site or not. To train the model, we use a portion of plip, obtained by filtering sequences appearing in our evaluation set and ProtBert context length (512 tokens).

Analyzing the results, we noted that RXNAAMapper generally manifests higher overlap scores and false positive rates in contrast to the supervised approach (Overlap Scores: $\mu_{RXNAAMapper} = 52.13\%$ and $\mu_{TokenClassification} = 41.84\%$. False Positive Rate: $\mu_{RXNAAMapper} = 47.89\%$ and $\mu_{TokenClassification} = 13.91\%$). Nonetheless, scrutinizing the distribution of distances between the predicted binding sites and the ground truth (see Figure 3), both methodologies showcased analogous outcomes, albeit the token classification approach displayed a marginally larger standard deviation ($\sigma_{RXNAAMapper} = 4.56$, $\sigma_{TokenClassification} = 10.87$, and $pvalue_{kstest} = 9.9 * 10^{-12}$) .

Based on these discernments, we infer that for cases where prior knowledge of the amino acid (AA) sequence is available, training a supervised model on similar protein data and employing it for binding site prediction confers distinct advantages. Conversely, in scenarios where the protein's origin remains elusive or the protein significantly diverges from well-characterized counterparts, adopting RXNAAMapper yields a more profound insight into the probable AAs implicated in the binding site.

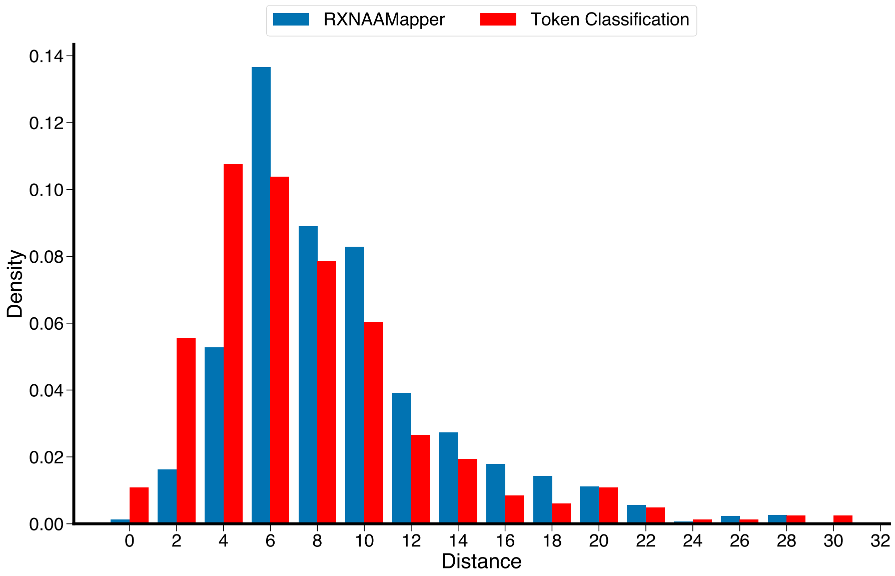

Figure 3: **Binding sites distance from ground truth**. This distribution plot illustrates the distances of the predicted binding sites obtained through RXNAAMapper and Token Classification approaches, with reference to the PLIP annotations. While both methods display comparable mean values, a notable difference emerges in their standard deviations. RXNAAMapper exhibits a tighter spread with $\mu = 9.99$ and $\sigma = 4.56$, while Token Classification presents a broader distribution with $\mu = 10.07$ and $\sigma = 10.87$.

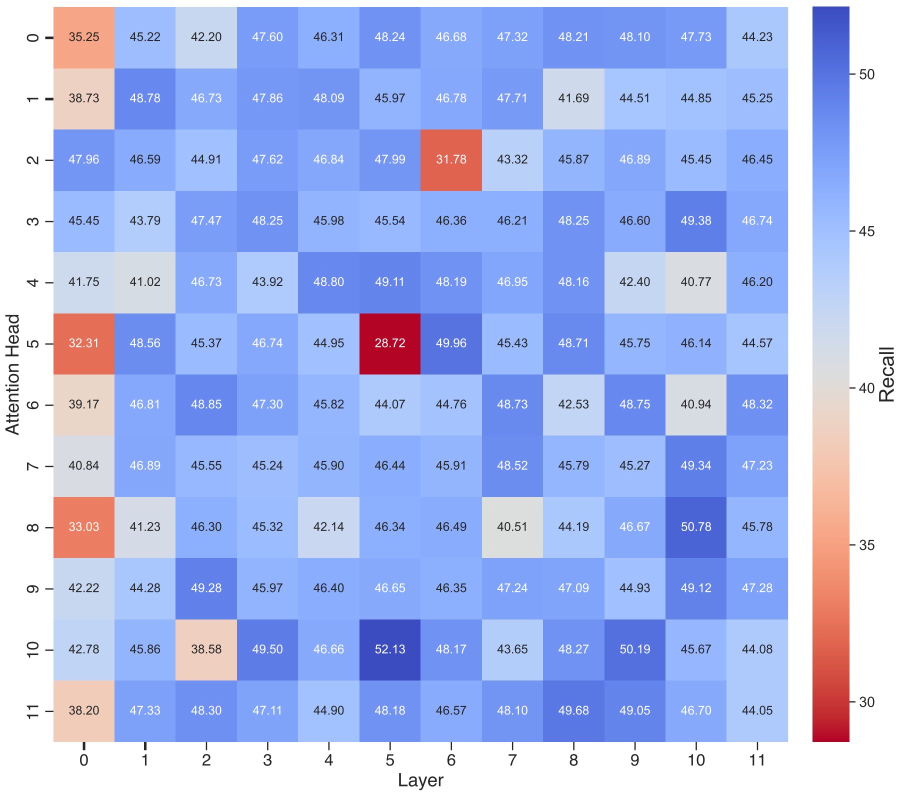

Figure 4: **Performance of the model evaluated by Recall**. Plotted across different layers and attention heads. The heatmap showcases the model's recall score for predicting binding sites, with the X-axis representing layers, the Y-axis representing attention heads, and the color bar indicating the recall score. Results are shown for the $top_k = 5$ setting, with optimal performance observed at head 10 and layer 5.

Complexity and scalability of RXNAAMapper

In this section, we present a detailed analysis comparing the computational efficiency of RXNAAMapper with other language models, including ProtAlbert, ProtBert, BERT-base, and BERT-large + BPE. Our investigation aims to demonstrate the competitive computational efficiency of RXNAAMapper, particularly in contrast to ProtAlbert and BERT-base. Our analysis reveals that RXNAAMapper exhibits significantly lower Floating Point Operations (FLOPs) compared to the other methods. Specifically, RXNAAMapper requires approximately 100 FLOPs, whereas ProtBert and BERT-large require around 330 and 420 FLOPs, respectively. This translates to a reduction of 70% and 76% in FLOPs compared to ProtBert and BERT-large, respectively. This efficiency advantage can be attributed to the streamlined tokenization strategies integrated into RXNAAMapper's architectural design, which are optimized for processing long sequences. By utilizing Byte Pair Encoding (BPE), RXNAAMapper reduces the token count required to represent protein sequences compared to traditional word-based vocabularies, leading to decreased model parameters and FLOPs. Furthermore, RXNAAMapper, based on BERT-base, features 12 layers as opposed to the 24 layers in BERT-large, resulting in 3.09 times fewer parameters for tuning compared to BERT-large. In comparison to ProtBert with 30 layers and 1024 hidden sizes, our model comprises 12 layers and a hidden layer size of 786. Computational efficiency is a crucial factor in practical applications, as it directly impacts the utilization of computational resources and runtime. A more efficient model like RXNAAMapper offers multiple benefits, including reduced computational burden, faster inference and training times, and enhanced scalability for integration into workflows involving large datasets or complex biological systems.

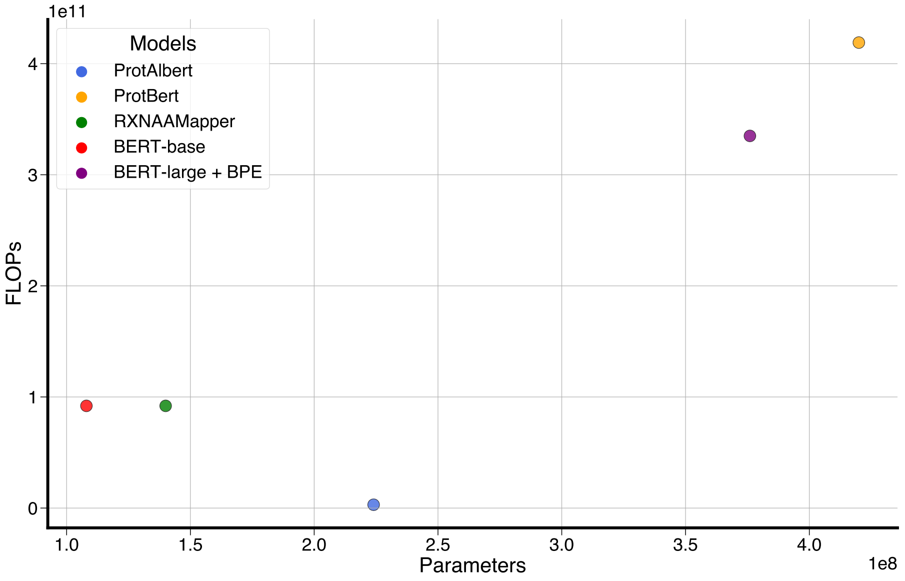

Figure 5: **Computational efficiency of RXNAAMapper compared to other language models**. This figure presents a comparative analysis of the computational efficiency of RXNAAMapper against other language models, such as ProtBERT and BERT-large. The x-axis depicts the number of parameters, while the y-axis represents the number of FLOPs required. The figure shows that RXNAAMapper exhibits a significantly lower number of parameters and FLOPs compared to models like ProtBERT, indicating its computational efficiency.
